# Two Aspects of Feedforward Control During a Fencing Lunge: Early and Anticipatory Postural Adjustments

**DOI:** 10.3389/fnhum.2021.638675

**Published:** 2021-06-14

**Authors:** Anna Akbaş, Wojciech Marszałek, Bogdan Bacik, Grzegorz Juras

**Affiliations:** Department of Human Motor Behavior, Institute of Sport Sciences, Academy of Physical Education, Katowice, Poland

**Keywords:** postural control, lunge initiation, center of pressure, base of support, temporal pressure

## Abstract

The present study investigated whether expertise in fencing influences the onset of postural preparation during the fencing lunge and how it changes under different performance conditions. We also questioned if the onset of feedforward control can be categorized into one of the postural phases: anticipatory or early postural adjustment. Eight elite fencers and nine physical education students performed an attack with a lunge in self-paced and reaction time conditions from three different initial stance widths. The onset of the center of pressure (COP) displacement and EMG activities for the tibialis anterior (TA) of both limbs were recorded. The results show that expertise in fencing delays the onset of the activity of TA of the front leg and the onset of COP displacement during fencing lunge performance in comparison to controls. Additionally, in contrast to the control group, fencers produce typical APA patterns in the activation of TA under different performance conditions, delayed reaction time in comparison to self-initiated lunging, and constant time of APA onset under different widths of stance. According to different times and functions of TA activity and COP displacement in lunging, we propose to address them as anticipatory postural adjustment and early postural adjustment, respectively.

## Introduction

The efficient control of body posture is fundamental to skillful sports performance. In fencing, fast and accurate decision-making in changeable combat conditions is considered as one of the key factors for success (Borysiuk and Waśkiewicz, 2008). The fencer should initiate the action as quickly as possible, all while maintaining the ability to execute effective movements regardless of the initial conditions. Therefore, to optimize fencing performance, it is crucial to assess how different performance conditions influence the phase of movement planning in terms of preparing one’s body posture for upcoming action.

It was first observed by Belenkiy et al. (1967) that during a rapid arm rise in a standing posture, the activation of the postural muscles precedes the onset of the voluntary movement (t_0_). Such an adjustment is a part of feedforward control and is referred to as anticipatory postural adjustments (APAs). APA can be observed up to 250 ms before upcoming action (Kanekar and Aruin, 2015), and its aim is to minimize the negative consequences of a disturbance on postural stability (Belenkiy et al., 1967; Massion, 1992). However, when the postural disturbance is associated with the displacement of the whole body, APA may play a role in generating forces that facilitate the execution of the movement (Bouisset and Do, 2008). Furthermore, APA may be also associated with stabilization of the given joint and reduce the number of redundant degrees of freedom (Bouisset and Zattara, 1987; Wang et al., 2018). Muscle activation during APA commonly induces the displacement of the center of pressure (COP) in the opposite direction to the direction of forthcoming disturbance (Belenkiy et al., 1967; Cordo and Nashner, 1982).

Anticipatory postural control is based on previous experience and can be acquired through learning (Aruin, 2016). It has been shown that APA can be trained in individuals with neurological disorders (Curuk et al., 2020), those with lower back pain (Tsao and Hodges, 2007; Brooks et al., 2012), those of older age (Aruin et al., 2015; Arghavani et al., 2020), and also healthy young adults (Saito et al., 2014; Aruin et al., 2015). According to the same authors, the training adaptations were associated with earlier onset of anticipatory muscle activity. The changes in the APA onset could be observed immediately after a single training session (Tsao and Hodges, 2007; Aruin et al., 2015; Kanekar and Aruin, 2015) or a set consisting of three trainings (Saito et al., 2014). According to Saito et al. (2014) the effect of repeated exercise was retained even after discontinuation of the training.

Despite the extensive knowledge of APA-based training in clinical practice, we are yet to determine whether anticipatory postural control can be mastered through long-term sports practice. It is widely accepted that elite athletes are characterized by more automated postural control in comparison to novices (Paillard, 2014; Michalska et al., 2018). More precisely, the differences in postural control measured by COP motion can be observed mostly in positions or tasks that are characteristic of a given discipline (Casabona et al., 2016; Paillard, 2019). As APAs are known to be task-specific and their improvement can be achieved by repeated practice of specific motor tasks, it is likely that long-term sports training would induce specific long-term adaptations in feedforward postural control of elite athletes.

It has been already reported in the literature that the APA might be altered throughout long-term training. For example, during preparation for unilateral leg movement, dancers were able to relocate their center of mass (COM) much faster than naive individuals. Subsequently, their movement required only a short adjustment in the final stage of COM displacement in comparison to controls (Mouchnino et al., 1992). In another study, untrained subjects were found to use twice the COM velocity and vertical-feet loading than trained subjects during raising from the chair task. In particular, the differences were observed only up to the point of seat-off (Cacciatore et al., 2014).

In several studies of postural control, another postural adjustment has been described called early postural adjustments (EPAs). EPA can be seen much earlier than classical APA (up to 500 ms before movement onset or the onset of postural disturbance), and its aim is to “ensure adequate mechanical conditions” for upcoming action (Krishnan et al., 2011a, 2012; Klous et al., 2012). Although EPA and APA differ both quantitatively and qualitatively, in most of the studies, they are considered to be one mechanism and are addressed as APA (Krishnan et al., 2011b; Klous et al., 2012; Bertucco et al., 2013).

There are two basic concepts about the occurrence of EPA. The first concept refers to the situation in which the person prepares the whole body for movement, such as taking a step. In this particular case, the EPA is considered as muscle activation that induces a shift of the COP beneath the feet backward and toward the initial swing limb (Klous et al., 2011; Krishnan et al., 2011a). The second concept says that EPA occurs at the early stage of motor planning and precedes the occurrence of APA. Therefore, it is possible to record both mechanisms as a sequence of events.

Although the influences of different environmental constraints on the APA onset are known in the literature, the issue of how the EPA changes under external constraints is unclear. Firstly, the onset of APA was found to occur later in self-paced (SP) and choice reaction time (CRT) conditions than in the movements performed under simple reaction time (SRT) instruction (De Wolf et al., 1998; Slijper et al., 2002). In contrast, the onset of EPA was constant in both SP and SRT when a person was preparing for whole-body postural sway (Klous et al., 2012), but they occurred later when the step was initiated after the acoustic stimulus in comparison to the same task under SP instruction (Yiou et al., 2015, 2016). Secondly, it has been proposed by Krishnan et al. (2012) that the presence of EPA in a given motor task may be associated with unnatural foot configuration, and, as a result, EPA may not be observed in a very comfortable position. It is important since we know, that EPA and APA may be registered sequentially in a single trial at their typical time intervals (Wang et al., 2006; Lee and Aruin, 2013; Ida et al., 2017). Nevertheless, if the change in position was associated with the change in the base of support (BOS) size, no changes in APA and EPA onset were found in pointing tasks and during gait initiation, respectively (Rocchi et al., 2006; Yiou and Schneider, 2007; Honeine et al., 2016). What is more, regardless of the change in initial position and BOS, the EPA was present during step initiation as the early shift of COP (Rocchi et al., 2006).

The fencing lunge, as a very complex sport-specific movement, is often compared to the arm pointing task followed by rapid stepping (Yiou and Do, 2000, 2001; Yiou and Schneider, 2007). It has been already shown in the literature that the fencing lunge was preceded by early COP backward displacement (Yiou and Do, 2001). In particular, the authors demonstrated that in contrast to novice, the experienced fencers were able to use APA in developing higher touch velocity. However, several issues concerning feedforward postural control in fencing lunge performance remain unclear.

Firstly, as the earlier onset of APA is associated with better postural preparation, we would like to assess whether expertise in fencing influences the onset of postural preparation (muscle activation and associated COP displacement) in comparison to non-fencers. Secondly, we would like to examine how performance conditions (reaction time instruction and width of stance) influence the onset of feedforward postural control in the fencing lunge. Thirdly, we would like to assess whether observed phenomena can be categorized (based on time intervals from the literature) into one of the postural control phases: early and anticipatory postural adjustment phase. We hypothesize that elite fencers will be characterized by earlier postural control onset than control subjects. Additionally, we suppose that the onset of postural adjustments under different performance conditions will change similarly to the step initiation. Particularly, APA will occur earlier under SP than reaction time instruction and will be time locked under different stance widths. At last, we suppose that the onset of feedforward postural control will occur in the early postural adjustment phase. To test our hypothesis we recorded muscles activity and COP displacement that precede the fencing lunge execution in two groups of subjects: elite fencers and controls familiar with the fencing lunge technique.

## Materials and Methods

### Subjects

Eight elite epee fencers, all members of the Polish National Team, and nine students of physical education participated in this study (Table 1). All subjects were women. In order to familiarize the technique of an attack with a lunge, students participated in three 30-minute training sessions carried out according to the adopted methodology by a fencing coach (Czajkowski, 2005). The subjects were included in the study if the fencing coach confirmed that the subjects were familiar with the adopted fencing lunge technique and they could perform an action correctly under SP, SRT, and CRT instruction. At last, all of the subjects who participated in the training were included in our study. The subjects provided their informed written consent for voluntary participation in the study. The study was approved by the Institutional Bioethics Committee.

**TABLE 1:** Physical characteristics of subjects.

	**N**	**Sex**	**Age**	**Height**	**Weight**
			** (mean ± SD)(years)**	**(mean ± SD)(cm)**	**(mean ± SD)(kg)**
Fencers	8	F	21.75 ± 3.27	173.56 ± 10.49	64.81 ± 8.62
Controls	9	F	21.4 ± 0.8	161.7 ± 6.07	58.49 ± 6.3

### Apparatus

Analog outputs (voltage) from two force platforms (AMTI, AccuGait, United States) were collected synchronously at 100 Hz sampling frequency using a 16-bits analog data acquisition device (Measurement Computing, USB-1616FS, United States). Then ground reaction forces and the associated moments were calculated using the calibration matrix and formulas provided by the manufacturer. Platforms were placed in line along the sagittal axis. Resultant COP in the AP direction (COP_*AP_res*_) was calculated taking into account the offset of the center of the rear platform relative to the center of the front platform, using the following formulas:

$$C\,O\,P_{A\,P\,\_\,1}=\frac{M_{x\,\_\,1}-\left(Z\,o\,f\,f\times F_{y\,\_\,1}\right)}{F_{z\,\_\,1}}$$

$$C\,O\,P_{A\,P\,\_\,2}=\frac{M_{x\,\_\,2}-\left(Z\,o\,f\,f\times F_{y\,\_\,2}\right)}{F_{z\,\_\,2}}$$

$$M_{x\,\_\,r\,e\,s}=C\,O\,P_{A\,P\,\_\,1}\times F_{z\,\_\,1}+\left(Y_{2}\,o\,f\,f+C\,O\,P_{A\,P\,\_\,2}\right)\times F_{z\,\_\,2}$$

$$C\,O\,P_{A\,P\,\_\,r\,e\,s}=\frac{M_{x\,\_\,r\,e\,s}}{F_{z\,\_\,r\,e\,s}}$$

where 1 and 2 are variables for the front and rear platform, respectively, and res means the resultant variables from both platforms. M_*x*_ is the moment of the force about the frontal axis, F_*y*_ is the horizontal component of the ground reaction force in the AP direction, and F_*z*_ is the vertical component of the ground reaction force. Zoff is the vertical offset from the top plate to the origin of the force platform, and Y_2_off is the offset of the center of the rare platform relative to the center of the front platform in the AP direction. COP_*AP_res*_ signal was filtered using a low-pass-4th-order Butterworth filter with a cut-off frequency of 7 Hz.

The electrical muscle activity of the tibialis anterior (TA) of both limbs was recorded at a sampling rate of 1,500 Hz using a wireless surface EMG system (Noraxon, Teleymo DTS Desk Receiver, United States), with a gain of 500, Common Mode Rejection Ratio (CMRR) greater than 100 dB and resolution of 16 bits. Disposable surface Ag/AgCl electrodes (Medtronic, H124SG Covidien Kendall, United States) were located according to the recommendations of SENIAM. The tibialis anterior was specifically selected due to its significant role in postural control during whole-body movement (Lepers and Brenière, 1995; Le Pellec and Maton, 2000; Aloraini et al., 2019) and fencing lunge execution (Borysiuk et al., 2014). The signal post-processing included band-pass filtration (10–500 Hz). Then the signal was rectified and filtered using a low-pass-2nd-order Butterworth filter with a cut-off frequency of 10 Hz in order to create a linear envelope.

The onset of the fencing lunge (t_0_) was registered using a wireless 3D accelerometer (Noraxon, DTS, United States) with a sampling rate of 1,500 Hz. According to the rules of the International Fencing Federation, an attack with a lunge must be preceded by upper limb forward progression. Several studies confirmed that fencing lunge starts with upper limb movement, however, not always in AP direction (Stewart and Kopetka, 2005; Szczygioł et al., 2016). Thus, to obtain t_0_ a 3D accelerometer was located on the wrist of the armed upper limb of the fencer. In order to create reaction time conditions, a module consisting of two lamps (red and yellow) was used in this study. All of the systems were synchronized using MaxPro Software v. 1.6.1.5 (Innovision-systems Inc.).

### Experimental Setup and Procedure

**FIGURE 1:**
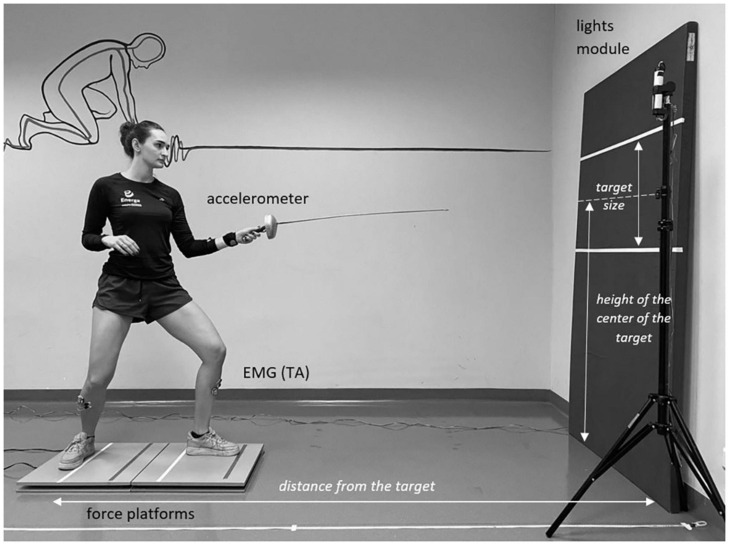
Experimental set-up.

Due to the fact that the *en garde* stance is relatively wide, we used two force platforms to provide sufficient space for testing for each subject. If necessary, the space between the platforms was filled with a wooden beam (12,5 cm wide). The placement of platforms in a line allowed the subjects to locate the front and rear feet on the front and rear platforms, respectively (Figure 1). A 50 cm high target was placed on a mattress. The width of the target was limited only by the edges of the mattress. The center of the target was placed at a height corresponding to 70% of the height of the subject. The distance from the target was also individualized and determined as 150% of the examined subject’s height (Gutiérrez-Dávila et al., 2014). The distance was measured from the mattress to the toes of the rear foot. All subjects were using an epee with a 90 cm long blade.

Subjects were instructed to perform an attack with a lunge as fast as possible to the target in nine experimental conditions. Each condition was defined by a change in the width of the subject’s initial position and reaction time manner. The preferred width of stance was assessed as the average distance between the heels in the subject’s natural *en garde* stance across three consecutive measurements. This distance was increased or decreased by 20% in order to change the size of the initial base of support. The reaction time conditions consist of simple reaction time (SRT), choice reaction time (CRT), and self-paced manner (SP). In SRT, the stimulus was provided by a light on the red lamp. In CRT, subjects had to perform an attack with a lunge immediately after the yellow lamp was lit and then lower the armed upper limb and point to the floor after the red lamp was lit. After a 10-minute standard warm up, the subjects executed combinations of the above-mentioned conditions in a randomized order:

• preferred, wide or narrow stance in an SP manner (10 valid trials each).
• ∂ preferred, wide or narrow stance in an SRT manner (10 valid trials each).
• ∂ preferred, wide or narrow stance in a CRT manner (20 valid trials each—10 red and 10 yellow).

The order of stimuli presentation in CRT was randomized. In the SRT and CRT conditions, the stimuli were presented after a time that allowed subjects to return to force platforms, adjust and stabilize their initial position, and declare their readiness for trial execution. After that, the stimuli were presented within 3 to 5 s. In SP, the time interval between trails was reduced to the time required to return to the initial stance. The trial was considered invalid when the subject missed the target or reacted incorrectly (i.e., performed an incorrect action in CRT condition or performed a false start—reacted in less than 100 ms). Invalid trials were not analyzed. To avoid the effects of fatigue, the time interval between conditions was 3 min.

### Data Processing

Data processing was conducted using Matlab software (Math Works Inc., R2017b). At first, the resultant acceleration from x, y, and z directions was calculated as the Euclidean norm and used to obtain t_0_. The onset of the movement was defined as the point that, in the researcher’s opinion, corresponds to the beginning of the upper limb movement (rapid change in resultant acceleration signal). After that, the average acceleration was calculated in a time period 1,000–1,300 ms before that point. Finally, the onset of upper limb movement defined by the researcher was corrected by an algorithm and corresponds to the point in which the value of acceleration exceeded ± 2.5 SD from the average and was maintained for at least 25 ms.

To check whether the upper limb movement always preceded lower limb movement, we compared the onset of t_0_ with the time of foot-off of the front lower limb. The foot-off was determined as the moment in which the vertical ground reaction force obtained from the front platform was equal to zero.

The time preceding the lunge initiation was divided into the (1) APA phase (up to 250 ms before t_0_) and (2) EPA phase (251–500 ms before t_0_). The onset of bioelectrical activation of the muscles was calculated from linear envelopes and corresponded to the point in which the envelope exceeded 2.5 SD (in corresponding time periods) from the average value in the time period 1,000–1,300 ms before t_0_ and maintained for at least 25 ms. Due to the high preliminary activation of the TA of the rear limb in the initial *en garde* stance, precise determination of the onset of electrical activity was possible only for TA of the front leg and six of eight experienced fencers. The onset of bioelectrical activity in control groups was determined for all of the subjects.

The onset of the COP displacement was determined based on the temporal velocity (vCOP) of the COPAP_res signal in which positive and negative values correspond to forward and backward movement, respectively. This point was determined as the last root in transition from positive to negative values of the vCOP (start of the backward movement) preceding t_0_.

### Statistical Analysis

As the onset of COP displacement was observed in the APA and EPA phases both, to compare the number of trials registered in the APA and EPA phases in both examined groups, the Chi-Square test was performed.

A three-way repeated-measures ANOVA with factors of two groups (two levels: fencers and control), the reaction time conditions (three levels: SP, SRT, and CRT), and different widths of stance (three levels: narrow, preferred, and wide) using a general linear model was performed to compare the onset of EMG activity and COP displacement, separately. For significant main effects, the Tukey’s *post hoc* analysis was conducted. The level of statistical significance was adopted for the value of *p* < 0.05. Statistical analyses were performed using Statistica v.13.3 (TIBCO Software Inc.).

## Results

The beginning of the upper limb movement (t_0_) preceded the foot-off of the front lower limb in fencers (mean 167 ms ± 68 SD) and controls (175 ms ± 114 SD).

### EMG Signal Analysis

**FIGURE 2:**
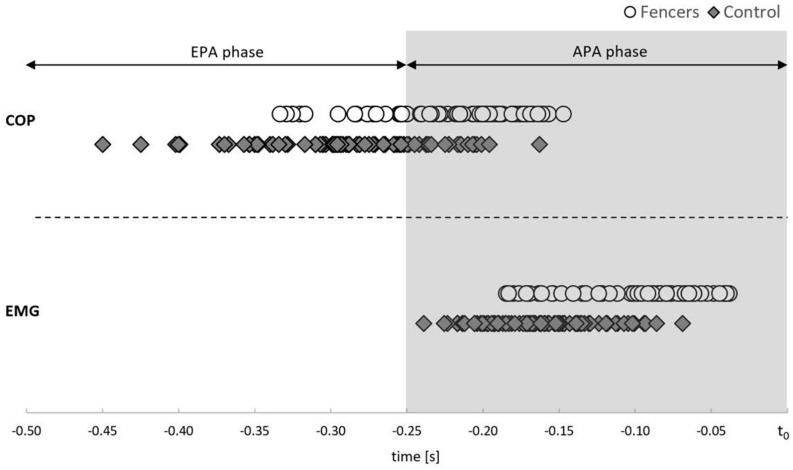
The average time of the onset of COP displacement (above dashed line) and the average time of the onset of TA activity (below the dashed line) in relation to movement onset (t_0_) for each subject in each experimental condition in fencers and controls. The shaded time interval corresponds to the APA phase, while the white time interval corresponds to the EPA phase.

**FIGURE 3:**
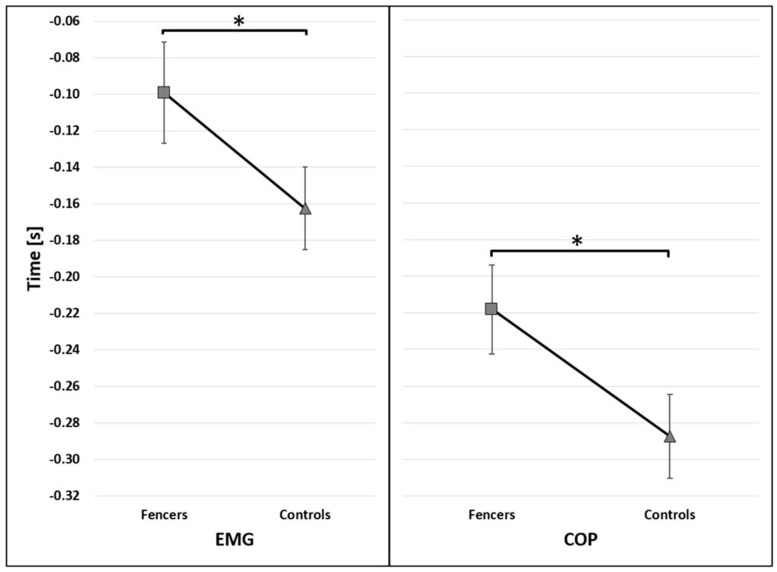
The average time of the onset of TA activity (EMG) and COP displacement (COP) before t_0_ for group factor (95% confidence interval presented as error bars). The significant differences between groups were marked as *.

Due to the high preliminary activity of TA of the rear limb, the results are presented only for TA of the front limb. The mean onset of bioelectrical activity of TA was registered in the APA phase (250 ms before t_0_) in both examined groups (Figure 2). The muscle activity of TA occurred earlier in fencers (mean 99 ms before t_0_) than in control group (mean 162 ms before t_0_) [*F*(1,13) = 14.527, *p* = 0.002, η^2^ = 0.528] (Figure 3).

**FIGURE 4:**
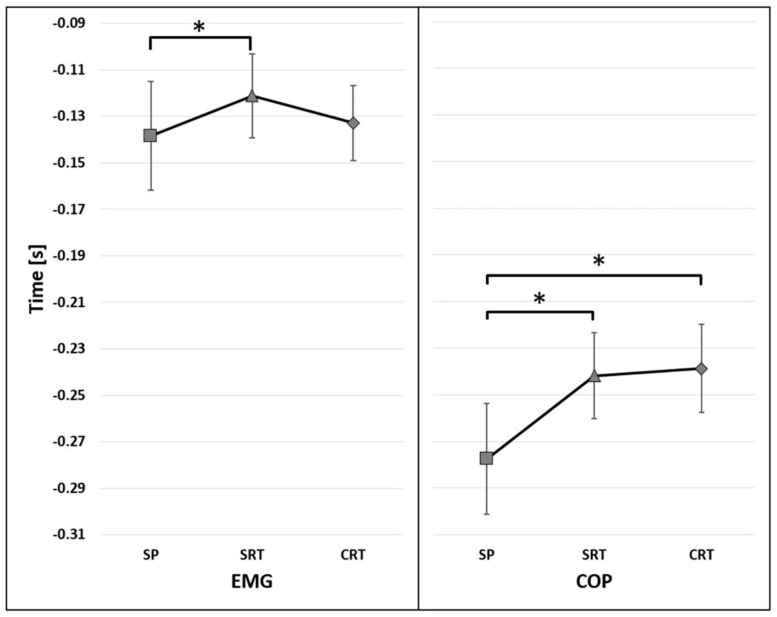
The average time of the onset of TA activity (EMG) and COP displacement (COP) before t_0_ for reaction factor (95% confidence interval presented as error bars). The significant differences between reaction time conditions were marked as *.

**FIGURE 5:**
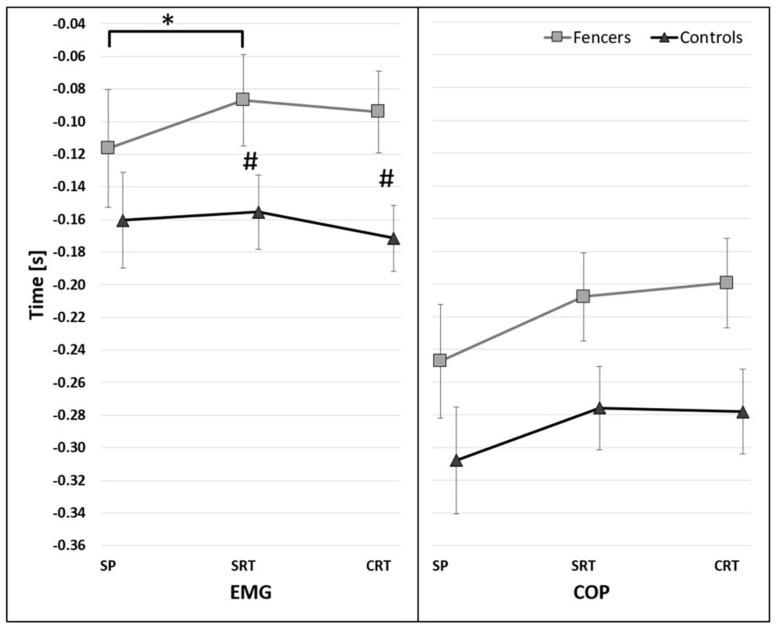
The average time of the onset of TA activity (EMG) and COP displacement (COP) before t_0_ for interaction between reaction and group factors. The significant differences between groups were marked as #. The significant differences between reaction time conditions were marked as * (95% confidence interval presented as error bars).

The significant main effect of reaction time conditions [*F*(2,26) = 4.376, *p* = 0.023, η^2^ = 0.252] on the onset of TA activity was found. The post-hoc analysis show significant differences between SP and SRT regardless initial stance condition (*p* = 0.045) (Figure 4). In addition, the significant effect of interaction has been found between the reaction and group [*F*(2,26) = 4.284, *p* = 0.024, η^2^ = 0.248], showing that the difference between groups were observed under SRT (*p* = 0.014) and CRT (*p* = 0.005) instruction (Figure 5).

No significant effects were found for position [*F*(2,26) = 0.853, *p* = 0.438, η^2^ = 0.062].

### COP Signal Analysis

The mean onset of COP displacement was registered in EPA, as well as in APA phase (mean for fencers 218 ms before t_0_; controls 287 ms before t_0_) (Figure 2). The onset of COP displacement occurred more often in EPA phase in controls than in fencers [narrow stance CRT: χ^2^ (1, 18) = 9.920, *p* = 0.002; preferred stance SRT: χ^2^ (1, 18) = 10.578, *p* = 0.001, CRT: χ^2^ (1, 18) = 8.242, *p* = 0.004; widest stance SP: χ^2^ (1, 18) = 4.897, *p* = 0.027, CRT: χ^2^ (1, 18) = 8.242, *p* = 0.004].

The onset of COP displacement occurred earlier in fencers (mean 218 ms before t_0_) than in controls (mean 287 ms before t_0_) [*F*(1,15) = 19.468, *p* < 0.001, η^2^ = 0.565] (Figure 3). The significant main effect of the reaction time condition [*F*(2,30) = 9.927, *p* < 0.001, η^2^ = 0.398] was found. The post-hoc analysis showed that under SP instruction the onset of COP displacement occurred earlier than in SRT (*p* = 0.003) and CRT (*p* = 0.001) (Figure 4).

No significant main effect of position [*F*(2,14) = 2.25, *p* = 0.142, η^2^ = 0.152] on onset of COP displacement was found.

## Discussion

The aim of the present study was to assess whether the long-term fencing training can change the time characteristic of feedforward postural control in fencing specific movement and how the onset of this control changes under different performance conditions. We also questioned if the onset of feedforward control can be categorized into one of the postural phases—anticipatory or early postural adjustment. The results show that the onsets of both TA activity and COP displacement were earlier in the control group than in fencers, but the differences between groups were significant under SRT and CRT in TA activity. The onset of both TA activity and COP displacement was influenced by reaction time conditions but not by the width of the initial *en garde* stance. In addition, the onset of bioelectrical activity of TA was always in the APA phase in both groups, while the onset of COP displacement occurred more often in the EPA phase in the control group and the APA phase in fencers.

The onset of TA activity and COP displacement occurred earlier in control subjects than elite fencers. This result did not support our hypothesis and is in direct contrast to previous studies investigating the effect of training on APA onset (Kanekar and Aruin, 2015; Aruin, 2016; Curuk et al., 2020). In our opinion, there are two possible explanations for that result. First is that the long postural preparation would not be efficient for fencers who are demanded to be performed under limited time and changeable combat situations. As it is known that the effect of motor experience on postural balance is very task dependent, we suppose that expertise in fencing (i.e., automatization in lunge performance) leads to specific adaptations in feedforward postural control and delay the onset of TA activity and COP displacement before lunging. In the literature, we found one example when the more skillful performance induced delayed feedforward control. In particular, the onsets of the EPA and APA were shifted toward t_0_ in young as compared to older subjects during gait initiation (Wang et al., 2016). Since the differences in the mean onset of postural control in examined groups were significant under SRT and CRT instruction, we are likely to accept another explanation. It has been proposed in the literature that the onset of feedforward postural control could be affected by actual reaction time. According to Slijper et al. (2002) the 1 ms increase in reaction time corresponded to the increase in the delay between APA and t_0_ onsets between 0.1 and 0.6 ms. Although we did not provide any data related to the reaction time, previous studies show significant shorter RTs in fencers when compared to control subjects (Borysiuk and Waśkiewicz, 2008).

The fact, that the differences between groups were more pronounced under RT conditions suits the idea that sport-specific training induces postural adaptations that are observed in the context in which they were trained (Paillard, 2017). Fencing, considered an open-skill sport discipline, requires fast and accurate decision-making as a response to the opponent’s actions. Therefore, the differences in postural adaptations between elite fencers and non-fencers were observed when the motor task (lunging) was performed under temporal pressure. The influence of evaluation conditions on postural adaptations was also found in the previous studies (Paillard et al., 2002; Asseman et al., 2008). For example, the postural performance of elite surfers was better than lower-class surfers only in dynamic conditions that were related to the sport-specific context, maintaining the stable posture regardless of the wave movement (Paillard et al., 2011).

The onset of TA activity of the front leg changed significantly under the reaction time conditions and was delayed under SRT and CRT as compared to SP lunge but occurred earlier in CRT than in SRT. Additionally, no effect of initial stance width was found. These findings support our hypothesis and are consistent with previous studies which examine APAs onset under different reaction time instructions and different base of support size (De Wolf et al., 1998; Slijper et al., 2002; Yiou et al., 2007; Kennefick et al., 2018). However, the dynamics of postural adjustment were different between the groups. In fencers, the onset of TA activation was significantly different under SP and SRT, while in controls no significant difference between reactions was observed. Although in general, the long-term fencing training shortens the phase of postural adjustment, it appears that fencers used a lack of time pressure to generate much longer adjustment.

The onset of COP displacement occurred earlier in SP compared to SRT and CRT. However, in SRT, the onset of COP displacement occurred earlier than in CRT. These results support our hypothesis are in line with previous studies in which postural control, defined as an early COP shift and body weight transfer, changed significantly under temporal pressure (Yiou et al., 2012, 2015, 2016; Hussein et al., 2013). The COP displacement duration was shortened in healthy subjects when instructed to take a step over an obstacle as soon as possible after hearing an acoustic stimulus (Yiou et al., 2015, 2016). The shortened phase of COP displacement under simple reaction time conditions was also observed during rapid leg flexion in healthy young subjects and rapid leg flexion and finger extension in response to an acoustic stimulus in elderly adults (Yiou et al., 2012; Hussein et al., 2013).

In our study COP displacement was observed in any case and changed similar to in step initiation process. However, no significant effect of position on the onset of COP displacement was found. These results are in line with the studies, which showed no effect of COP displacement onset under different stance widths (Rocchi et al., 2006; Yiou et al., 2007; Honeine et al., 2016). Honeine et al. (2016) showed no effect of the mediolateral COP displacement duration when the gait was initiated from three different stance widths corresponding to 15, 30, and 45 cm. However, the initial width of the stance influenced the amplitude of these adjustments. Similar results were found by Rocchi et al. (2006) investigating the influence of initial stance conditions on healthy adults and patients with Parkinson’s disease.

Our results show the onset of activity of TA of the front leg was always after the onset of COP displacement. Due to the fact, that the movement is a natural consequence of internal and external forces acting on the human body, the onset of COP displacement should be observed slightly after the muscle activation (Loram and Lakie, 2002). For example, in gait initiation, the alternating EMG activity of the TA and SOL results in shifting the COP both backward and toward the initial swing limb (Brenière et al., 1987). This allows the production of forces that propel the body forward and toward single leg support, reaching the desired gait velocity (Lepers and Brenière, 1995). However, in our study, the COP displacement could not be caused by activation of TA of the front leg, and, consequently, the early shift of COP must be induced by the activation of other muscles. As the propulsive phase of the lunge is mainly associated with the rear but not the front leg and the movement velocity is correlated with the activity of the gluteus maximus and vastus lateralis of the rear limb, the change of rear limb muscle activation might contribute to early COP shift (Guilhem et al., 2014; Chen et al., 2017). Nevertheless, if TA of the front leg was the first active lower limb muscle, the early COP displacement must be initiated in muscles of the torso. Based on our results, we cannot also exclude that the movement was generated in another postural muscle (i.e., gastrocnemius medialis and laterialis) that is, next to TA and SOL, of primary importance during standing (Vieira et al., 2012; Héroux et al., 2014). Therefore, the topic of postural muscle contribution in COP displacement in fencing lunge requires further investigation.

As the TA activation did not contribute to COP displacement, we suppose that the main goal of TA activation was to stabilize the ankle joint. This idea is in line with the definition of APA, which points out, that the muscle activation during the APA phase may act to decrease the intersegmental movement and stabilize the joint (Wang et al., 2018). Hence, according to the muscular coordination pattern in lunging provided by Guilhem et al. (2014), it is known that TA of the front leg participates in the dorsiflexion of the ankle to stabilize the front leg during the lunge in fencing. In contrast, the same study showed that TA of the front leg is activated as the first lower body muscle during lunging and, as the muscle which crosses the ankle joint, contributes to movement initiation (Guilhem et al., 2014).

At last onset of TA activity occurred always in the APA phase (up to 250 ms before t_0_) while the onset of COP displacement was not clearly categorized into one of the feedforward control phases. The onset of COP displacement in the control group was shifted more into the EPA phase, while in fencers this occurred in the “early” APA and “late” EPA phase (around 150–350 ms before t_0_). Although the given time intervals are conventional, they are commonly used in the literature to distinguish both mechanisms (Lee and Aruin, 2013; Wang et al., 2016).

We suppose that the activity of TA and COP displacement were different in nature because of several reasons. Unlike we expected, the activity of TA did not generate the COP displacement. Additionally, the muscles which generated the COP displacement had to be activated much earlier than TA—in an earlier phase of motor planning. Finally, the adjustment observed as the COP signal—a shift in the COP backward to the rear limb—aimed to create optimal mechanical conditions for effective lunge performance, i.e., with a given speed and without balance loss while TA activity was rather associated with stabilization of ankle joint. Although we can address both variables as APA, due to their different function in lunging, we would like to address them as early postural adjustment (COP displacement) and anticipatory postural adjustment (TA activity). Such a distinction between these two mechanisms, according to their function and timing, is in line with previous studies (Krishnan et al., 2011b; Klous et al., 2012; Bertucco et al., 2013). Furthermore, according to Krishnan et al. (2011b), the adjustments that occur in the EPA phase cannot act against the disturbance but represents the balance interruption itself. Therefore, this cannot be considered to be “classical” of APA. However, the topic of the role of both mechanisms in lunging requires further investigation.

At last, the number of potential limitations and research perspectives need to be considered. Firstly, the examined movement of fencing technique—an attack with a fencing lunge—was very complex in its structure and required a movement sequence that starts by the extension of an armed upper limb followed by lower limb movement. Thus, we decided to mark the beginning of the lunge as upper limb motion and not at the point of heel off or toes off. Our results are encouraging and should be validated by the t_0_, which is determined as the movement of the lower limb. Moreover, it would be interesting to determine the postural control associated with upper and lower limb movement separately. Secondly, we recorded the activity of TA of the front leg, which did not contribute to the EPA phase. It is recommended that further research be undertaken in the area involving the trunk and thigh muscles. The present study is the first step toward enhancing our understanding of postural mechanisms in sports practice. To further our research, we plan to investigate the influence of early and anticipatory postural adjustments on the effectiveness of competitive fencers in terms of speed, accuracy, and decision-making.

## Conclusion

Long-term fencing training delays the onset of the activity of TA of the front leg and the onset of COP displacement during fencing lunge performance in comparison to controls. Additionally, in contrast to the control group, fencers produce a typical APA pattern in the activation of TA under different performance conditions—delayed under reaction time in comparison to self-initiated lunging and constant time of APA onset under different widths of stance. According to different times and functions of TA activity and COP displacement in lunging, we propose to address them as anticipatory postural adjustment and early postural adjustment, respectively.

## Data Availability Statement

The raw data supporting the conclusions of this article will be made available by the authors, without undue reservation.

## Ethics Statement

The studies involving human participants were reviewed and approved by Institutional Bioethics Committee of Academy of Physical Education in Katowice. The patients/participants provided their written informed consent to participate in this study. Written informed consent was obtained from the individual(s) for the publication of any potentially identifiable images or data included in this article.

## Author Contributions

AA contributed with the project creation, data collection, data analysis, and drafted the manuscript. WM contributed in the data collection and data analysis. BB contributed with the project creation and data analysis. GJ contributed with the project creation and manuscript preparation. All authors discussed the results and participated in the revision of the manuscript.

## Conflict of Interest

The authors declare that the research was conducted in the absence of any commercial or financial relationships that could be construed as a potential conflict of interest.
